# Amino Acids in the Uterine Luminal Fluid Reflects the Temporal Changes in Transporter Expression in the Endometrium and Conceptus during Early Pregnancy in Cattle

**DOI:** 10.1371/journal.pone.0100010

**Published:** 2014-06-24

**Authors:** Niamh Forde, Constantine A. Simintiras, Roger Sturmey, Solomon Mamo, Alan K. Kelly, Thomas E. Spencer, Fuller W. Bazer, Pat Lonergan

**Affiliations:** 1 School of Agriculture and Food Science, University College Dublin, Dublin, Ireland; 2 Center for Cardiovascular and Metabolic Research, University of Hull, Hull, United Kingdom; 3 Department of Animal Sciences, Washington State University, Pullman, Washington, United States of America; 4 Department of Animal Science, Texas A&M University, College Station, Texas, United States of America; The University of Georgia, United States of America

## Abstract

In cattle, conceptus-maternal interactions are critical for the establishment and maintenance of pregnancy. A major component of this early interaction involves the transport of nutrients and secretion of key molecules by uterine epithelial cells to help support conceptus development during the peri-implantation period of pregnancy. Objectives were to: 1) analyze temporal changes in the amino acid (AA) content of uterine luminal fluid (ULF) during the bovine estrous cycle; 2) understand conceptus-induced alterations in AA content; 3) determine expression of AA transporters in the endometrium and conceptus; and 4) determine how these transporters are modulated by (Progesterone) P4. Concentrations of aspartic acid, arginine, glutamine, histidine, lysine, isoleucine, leucine, phenylalanine and tyrosine decreased on Day 16 of the estrous cycle but increased on Day 19 in pregnant heifers (P<0.05). Glutamic acid only increased in pregnant heifers on Day 19 (P<0.001). Asparagine concentrations were greater in ULF of cyclic compared to pregnant heifers on Day 7 (P<0.05) while valine concentrations were higher in pregnant heifers on Day 16 (P<0.05). Temporal changes in expression of the cationic AA transporters *SLC7A1 SLC7A4* and *SLC7A6* occurred in the endometrium during the estrous cycle/early pregnancy coordinate with changes in conceptus expression of *SLC7A4, SLC7A2* and *SLC7A1* (P<0.05). Only one acidic AA transporter (*SLC1A5*) increased in the endometrium while conceptus expression of S*LC1A4* increased (P<0.05). The neutral AA transporters *SLC38A2* and *SLC7A5* increased in the endometrium in a temporal manner while conceptus expression of *SLC38A7*, *SLC43A2*, *SLC38A11* and *SLC7A8* also increased (P<0.05). P4 modified the expression of *SLC1A1*, *-1A4*, *-1A5*, *-38A2*, *-38A4*, *-38A7*, *-43A2*, *-6A14*, *-7A1*, *-7A5* and *-7A7* in the endometrium. Results demonstrate that temporal changes in AA in the ULF reflect changes in transporter expression in the endometrium and conceptus during early pregnancy in cattle, some of which are modified by P4.

## Introduction

A major cause of infertility in cattle is embryo mortality due to conceptus and/or uterine dysfunction during the pre-implantation period of pregnancy. In cattle, the majority of embryonic loss occurs prior to maternal recognition of pregnancy [1] which occurs on approximately Day 16 of pregnancy [2], [3]. Thus, conceptus-maternal interactions are critical to the establishment and maintenance of pregnancy. Major components of this early conceptus-maternal interaction involve transporters for nutrients and secretions of key molecules by uterine epithelial cells that support conceptus development during the peri-implantation period of pregnancy [4], [5], [6], [7]. These secretions and transported molecules make up histotroph that represents maternal contributions to uterine luminal fluid (ULF). The requirement for these uterine-derived secretions and transported nutrients for successful pregnancy is well established in the ovine uterine gland knockout model in which the conceptus fails to elongate [8]. In cattle, ULF is a prerequisite for development of the embryo beyond the hatched blastocyst stage in vivo, and attempts to artificially induce elongation of bovine conceptuses in vitro have been unsuccessful [9], [10]. The ULF of ruminants is composed of both secreted and transported molecules that include numerous secreted proteins [11], [12], glucose [13], ions [13], fatty acids [14] and amino acids [13], [15]. In sheep, a significant increase in total amino acid content occurs between Days 10 and 16 of pregnancy [13]. Moreover, the expression of cationic [16], acidic and neutral amino acid transporters is altered in a spatial and temporal manner in both the endometrium and conceptus of sheep during early pregnancy [13] and is modulated by ovarian progesterone (P4) and/or conceptus interferon tau (IFNT), prostaglandins and cortisol in vivo [17], [18], [19].

In spite of the importance of maternally-derived secretions, including amino acids, there are limited reports pertaining to the requirements for conceptus elongation and successful pregnancy recognition in cattle. What is known, however, is that amino acids are utilized by the early embryo both in vitro and in vivo [20], [21], [22], [23]. In addition, total amounts of amino acids increase in ULF as the estrous cycle progresses [24] and are higher in ULF of pregnant compared to non-pregnant heifers on Day 18 [12]. Furthermore the abundance of individual amino acids (e.g., valine) is regulated by P4 [25].

The hypothesis tested was that the amino acid composition of bovine ULF changes in a temporal manner during the estrous cycle and early pregnancy due to alterations in the expression of their transporters in the endometrium and conceptus. The objectives were to: 1) analyze the temporal changes in the amino acid content of ULF during the bovine estrous cycle; 2) understand conceptus-induced alterations in amino acid content of ULF during critical windows of early pregnancy; 3) determine expression of the transporters in the endometrium and conceptus responsible for shuttling these amino acids into and out of the uterine lumen; and 4) determine how these transporters are modulated in the endometrium by P4.

## Materials and Methods

All experimental procedures involving animals were licensed by the Department of Health and Children, Ireland, in accordance with the Cruelty to Animals Act (Ireland 1876) and the European Community Directive 86/609/EC and were sanctioned by the Animal Research Ethics Committee of University College Dublin. Unless otherwise stated, all chemicals were sourced from Sigma (Dublin, Ireland).

### Study 1: Analysis of the amino acid content of uterine luminal fluid and expression of amino acid transporters in the endometrium during the estrous cycle and peri-implantation period of early pregnancy

The estrous cycles of 100 cross-bred beef heifers were synchronized as previously described [26] by insertion of a controlled internal drug release (CIDR) device (1.38 g progesterone; InterAg, Hamilton, New Zealand) into the vagina for 8 days. A 2 ml intramuscular injection of a prostaglandin F_2α_ (PG) analogue (Estrumate, Shering-Plough Animal Health, Hertfordshire, UK: equivalent to 0.5 mg cloprostenol) was administered one day before CIDR removal. Heifers were checked for estrus and only those detected in standing heat (estrus = Day 0) were utilized further. In order to generate pregnant (P) and cyclic (C) tissues, heifers were assigned randomly to either an inseminated group (n = 59) or a non-inseminated cyclic control group (n = 24). Cyclic heifers were slaughtered on Day 7, 10, 13, or 16 of the estrous cycle and inseminated heifers were slaughtered on Day 7, 10, 13, 16 or Day 19 of pregnancy. These stages correspond in pregnant animals to the times of blastocyst formation, blastocyst hatching, initiation of elongation, maternal recognition of pregnancy and initiation of implantation, respectively. Cyclic heifers were not included for the Day 19 time-point as they had undergone luteolysis at this stage. At slaughter, the uterine horn ipsilateral to the corpus luteum (CL) was flushed with 20 ml of 10 mM Tris (pH 7.2) and in the inseminated group, only those flushings that contained an appropriately developed conceptus (i.e., correct stage for age) were processed further. Recovered ULF was centrifuged at 1,000 g for 15 min, supernatant decanted and immediately snap frozen in 1 ml aliquots in liquid nitrogen for subsequent amino acid analysis. Intercaruncular and caruncular endometrial tissues were dissected out separately from the uterine horn ipsilateral to the CL and snap-frozen in liquid nitrogen for RNA extraction and quantitative real-time PCR (qRT-PCR).

### Study 2: Expression of amino acid transporters in the conceptus during key developmental stages

Analysis of the expression of amino acid transporters in the embryo/conceptus was carried out by screening RNA sequencing data generated as previously described [27]. Briefly, estrus synchronization of cross-bred beef heifers (n = 72) was performed as described for Study 1. Heifers observed in standing heat were inseminated. Animals were assigned randomly for slaughter on Day 7, 10, 13, 16 or 19 of pregnancy. At slaughter, each uterine horn was flushed with 20 ml phosphate buffered saline (PBS) containing 3% fetal calf serum (FCS), the embryo/conceptus recovered and snap frozen in liquid nitrogen. Only those uterine flushings with conceptuses at the correct morphological stage of development for their age were analysed. RNA was extracted from whole conceptuses, cDNA libraries were prepared and cluster generation and sequencing were carried out using standard procedures for the Illumina genome analyzer sequencer (www.illumina.com). The RNAseq samples were processed through the standard software pipeline for the Genome Analyzer (http://bioinfo.cgrb.oregonstate.edu/docs/solexa/SCS2_01_IPAR1_01_Release_Notes.pdf). The CASAVA module from Illumina software was used to process RNAseq data. All data were aligned against the BosTau4 genome and a pseudochromosome containing potential splice junction sequences was generated. This gene expression data set was then screened for expression of members of the solute-like carrier (SLC) gene family for transport of amino acids.

### Study 3: Identification of changes in endometrial expression of amino acid transporters by manipulation of P4 concentrations in vivo

The estrous cycles of cross-bred beef heifers were synchronized as described in Study 1 and only those observed in standing estrus (n = 52) were used. Heifers were assigned randomly to one of three treatments, (i) high P4 (n = 12), (ii) normal P4 (n = 12) and (iii) low P4 (n = 28). Heifers in the high P4 group had a progesterone-releasing intravaginal device (PRID, CEVA, Libourne, France) inserted on Day 3 of the estrous cycle to elevate P4 concentrations [28], while heifers in the control group received no P4 manipulation. Heifers assigned to the low P4 group received three intramuscular injections of PG (Estrumate, Shering-Plough Animal Health, Hertfordshire, UK) on Day 3, 3.5 and 4 of the estrous cycle to reduce P4 output from the CL, as previously described [29]. Daily blood samples were obtained from all heifers up to day of slaughter on either Day 7 or Day 13 of the estrous cycle. Following slaughter, intercaruncular endometrial tissue from the tip of the uterine horn ipsilateral to the CL was recovered, snap frozen in liquid nitrogen for subsequent RNA extraction and qRT-PCR was performed for selected amino acid transporters.

#### Analysis of uterine luminal fluid amino acids

The amino acid content of ULF was measured by High Performance Liquid Chromatography (HPLC) as previously described [23]. In summary, the amino acids present in ULF were derivatised with *O*-Phthaldialdehyde reagent, supplemented with 1 mg/ml 2-mercaptoethanol. Reverse phase chromatography was subsequently performed on an Agilent 1100 Series HPLC system coupled with a Phenomenex HyperClone 5 mm C-18 ODS 250×4.6 mm column (Phenomenex, Macclesfield, UK). A gradient elution with two buffers comprised of (A) 80% 83 mM sodium acetate, 19.5% methanol, 0.5% tetrahydrofuran, and (B) 80% methanol and 20% 83 mM sodium acetate was used to separate OPA-amino acid derivatives at 30°C for 60 min at a flow rate of 1.3 ml/min. Concentrations (µM) were determined by comparing the area under the curve for each peak to those given from certified standards.

#### Quantitative real-time PCR analysis (qRT-PCR)

For both Studies 1 and 3 total RNA was extracted from 100 mg of endometrial tissue using Trizol reagent as per manufacturer's instructions. RNA clean-up and on-column DNase treatment were performed (Qiagen, Crawley, Sussex, UK). Both the quality and quantity of extracted RNA was determined using the Agilent Bioanalyzer (Agilent Technologies, Santa Clara, CA, USA) and Nanodrop 1000 (Thermo Fischer Scientific, DE, USA), respectively. One microgram of total RNA was converted to complementary DNA (cDNA) using Superscript III (Applied Biosystems, Foster City, CA, USA) and random hexamers as per manufacturer's instructions. All primers were designed using Primer-BLAST software (www.ncbi.nlm.nih.gov/tools/primer-blast/) to span exon-exon boundaries where possible. Each qRT-PCR reaction was carried out on the 7500 Fast Real-Time PCR System (Applied Biosystems) with 50 ng cDNA, optimized primer concentrations (Table S1), and 7.5 µl FAST Sybrgreen mastermix (Applied Biosystems) in a final reaction volume of 15 µl. Cycling conditions were as follows: 2 min at 50°C, 10 min at 95°C, and 40 cycles of 95°C for 15 sec and 60°C for 1 min and were carried out with the inclusion of a dissociation curve to ensure specificity of amplification. A standard curve was included for each gene of interest as well as for the normalizer gene to obtain primer efficiencies. All raw cycle threshold values were then imported into qbase^plus^ software (Biogazelle, Zwijnaarde, Belgium) where data were calibrated, normalized and expression values for each gene were determined in arbitrary units (CNRQ).

#### Data analysis

All data were analysed using the SAS statistical package (SAS Institute Inc., Cary, NC, version 9.1.3). For gene expression analysis, the log of the calibrated, normalized, relative expression values (CNRQ) in arbitrary units from the endometrium or transcripts per million from the embryo/conceptus were inputted into the SAS program while total concentrations of amino acids in the ULF were used for amino acid analysis. Data were checked for normality and homogeneity of variance by histograms, qqplots, and formal statistical tests as part of the UNIVARIATE procedure of SAS. Data that were not normally distributed were transformed by raising the variable to the power of lambda. The appropriate lambda value was obtained by conducting a Box-Cox transformation analysis using the TRANSREG procedure of SAS. The transformed data were used to calculate P values. Gene expression values were analyzed using the general linear model procedures (PROC GLM) with day, pregnancy status, P4 concentration and/or tissue type (i.e., caruncular vs intercaruncular) where appropriate as the main effects. Treatment effects on gene expression were separated by Tukey's test and a p value of ≤0.05 was considered significant. Concentrations of amino acids were analyzed with the MIXED procedure of SAS. Fixed effects included experimental treatment (cyclic and pregnant), day, and their interaction. The interaction term, if not statistically significant (P>0.10), was subsequently excluded from the final model. Heifer within treatment was included as a random effect. The type of variance-covariance structure used was chosen depending on the magnitude of the Akaike information criterion for models run under compound symmetry, unstructured, autoregressive, or Toeplitz variance-covariance structures. Differences between treatments were determined by F-tests using Type III sums of squares. The PDIFF command incorporating the Tukey test was applied to evaluate pairwise comparisons between treatment means.

## Results

### Amino acid content of ULF during the peri-implantation period of pregnancy in cattle

Analysis of the ULF detected the presence of 18 amino acids of which threonine and glycine were most abundant on all days examined. The acidic amino acids, aspartic and glutamic acids, were detected in the ULF throughout the estrous cycle and early pregnancy. Aspartic acid concentrations decreased on Day 16 in both pregnant and cyclic heifers compared to all other days; however, on Day 19 of pregnancy, concentrations were higher (P<0.05) than for Day 16 (Table 1). Glutamic acid concentrations did not change between Days 7 and 16 (P>0.05), but increased from Day 16 to Day 19 in pregnant heifers (P<0.001).

**Table 1:** The effect of day and pregnancy status on the abundance of amino acids (µM) in bovine uterine luminal fluid during the peri-implantation period of pregnancy on Days 7, 10, 13, 16 for cyclic heifers and Days 7, 10, 13, 16 and 19 for pregnant heifers (n = 5 per treatment per time-point).

Amino acid	Treatment	Day 7	Day 10	Day 13	Day 16	Day 19	Day effect	Pregnancy effect	Day x Pregnancy
***Basic amino acids***									
Arginine	Cyclic	12.34±2.77ax	8.34±2.48ax	13.32±2.48ax	7.31±2.77bx	-	***	*	ns
	Pregnant	7.89±2.26ay	5.97±2.26ay	7.32±2.26ay	7.14±2.77ax	15.94±7.15b			
Histidine	Cyclic	5.67±1.21ab	4.23±1.21ab	5.79±1.21a	2.37±1.21b	-	ns	ns	ns
	Pregnant	3.69±0.99a	4.26±0.99a	5.01±1.40ab	4.33±1.21a	8.54±2.88b			
Lysine	Cyclic	16.38±2.92a	9.78±2.90ab	13.57±2.61a	5.58±1.75b	-	ns	ns	ns
	Pregnant	10.38±2.95a	7.23±2.76a	8.43±1.77a	9.55±10.70a	21.47±8.51b			
***Acidic amino acids***									
Aspartic acid	Cyclic	12.52±2.92a	12.62±2.61a	12.25±2.61a	4.50±2.92b	-	*	ns	ns
	Pregnant	9.08±2.38a	9.70±2.38a	8.93±2.38a	4.13±2.92b	9.76±4.37a			
Glutamic acid	Cyclic	47.11±10.66a	43.84±9.54a	43.54±10.74a	22.06±16.60a	-	ns	ns	ns
	Pregnant	38.58±7.60a	39.53±4.72a	40.40±7.74a	21.76±2.42a	69.43±27.18b			
***Small neutral amino acids***									
Alanine	Cyclic	35.27±8.09a	39.35±7.24a	42.54±7.24a	23.90±8.09a	-	ns	ns	ns
	Pregnant	26.63±6.61a	27.54±6.61a	30.28±6.61a	30.59±8.09a	51.13±17.99a			
Asparagine	Cyclic	3.12±0.35ax	1.63±0.35b	1.44±0.31b	0.80±0.40b	-	***	ns	0.1
	Pregnant	2.14±0.29ay	1.33±0.29b	1.28±0.40ab	1.68±0.35ab	3.41±1.47ab			
Glycine	Cyclic	114.12±42.78a	150.84±92.73a	65.91±28.07a	52.35±30.50a	-	ns	ns	ns
	Pregnant	129.90±57.83a	133.98±77.48a	57.41±36.66a	30.41±6.00a	54.78±22.77a			
Serine	Cyclic	21.63±3.94a	20.42±3.52a	17.27±4.55a	14.89±3.94a	-	ns	ns	ns
	Pregnant	15.78±3.22a	13.13±3.22a	22.23±3.52a	22.90±3.94a	27.64±9.26a			
Threonine	Cyclic	177.03±14.41ax	268.62±12.89bx	213.52±12.89ax	181.33±14.41ax	-	***	0.06	***
	Pregnant	208.61±11.77ax	195.70±12.89ay	212.91±11.77ax	151.07±14.41bx	124.53±5.96b			
***Large neutral amino acids***									
Glutamine	Cyclic	24.37±4.25ax	17.08±4.45abx	24.18±4.17ax	12.74±3.74bx	-	***	***	***
	Pregnant	14.60±7.07ax	16.47±7.45ax	21.97±8.20ax	18.17±1.62by	36.79±11.89c			
Isoleucine	Cyclic	8.81±1.58a	5.87±1.41ab	7.36±1.41ab	3.27±1.58b	-	ns	ns	ns
	Pregnant	5.79±1.29a	4.45±1.29a	4.39±1.29a	5.15±1.58a	9.78±3.82b			
Leucine	Cyclic	17.60±3.26a	12.27±2.91ab	15.73±2.91a	6.57±3.26b	-	ns	ns	ns
	Pregnant	11.78±2.66a	9.18±2.66a	9.73±2.66a	10.45±3.26a	20.28±9.32b			
Methionine	Cyclic	10.98±1.68ax	11.83±1.94ax	5.92±1.50bx	2.85±1.68bx	-	***	*	ns
	Pregnant	7.83±1.37ax	6.97±1.37aby	4.48±1.37abx	3.43±1.68bx	7.66±3.39a			
Phenylalanine	Cyclic	7.46±1.22a	5.84±1.22ab	6.14±1.09a	2.77±1.22b	-	ns	0.07	ns
	Pregnant	5.14±1.00a	3.69±1.00a	3.59±1.00a	4.59±1.22a	9.48±3.82b			
Tryptophan	Cyclic	3.46±0.83a	3.42±0.83a	2.89±0.96a	1.23±1.18a	-	ns	ns	ns
	Pregnant	2.03±0.68a	2.65±0.68a	2.03±0.96a	2.43±0.96a	5.49±2.64b			
Tyrosine	Cyclic	7.55±1.45a	6.06±1.45ab	6.15±1.30ab	2.66±1.45b	-	ns	ns	ns
	Pregnant	4.82±1.19a	5.41±1.19a	3.66±1.19a	4.43±1.45a	8.45±3.14b			
Valine	Cyclic	15.57±3.90ax	10.07±3.81ax	13.15±2.97ax	6.33±0.00bx	-	***	ns	***
	Pregnant	10.88±1.08ax	8.81±0.66bx	7.89±0.53bx	9.31±0.00cy	17.10±6.31d			

Significant overall effects of day, pregnancy status and their interactions are noted by an asterisk (*). Temporal differences are indicated by different superscript letters a, b, c, i.e. significant differences in amino acid abundance between sequential days of the estrous cycle or early pregnancy when P<0.05. Differences between pregnant and cyclic heifers on a given day are denoted by x,y when P<0.05.

The basic amino acids arginine, histidine and lysine, displayed similar changes in abundance as the concentration of all these amino acids decreased on Day 16 of the estrous cycle compared to other days of the estrous cycle. This decrease did not occur in pregnant heifers; indeed, the concentrations of all three basic amino acids in ULF was greater on Day 19 compared to Day 16 (P<0.05).

Of the small neutral amino acids, concentrations of alanine, glycine and serine in ULF from both pregnant and cyclic heifers were similar for all days examined (P>0.05). Concentrations of asparagine were highest in cyclic heifers on Day 7, declined significantly by Day 10 and remained low thereafter, while in pregnant heifers, despite an initial decline in concentrations on Day 10, concentrations were relatively stable throughout early pregnancy. Concentrations of asparagine were greater in ULF of cyclic compared to pregnant heifers on Day 7 (P<0.05). Concentrations of threonine were elevated on Day 10 compared to all other days in cyclic heifers while concentrations in pregnant heifers were lower (P<0.05) on Day 16 and 19 compared with earlier time-points.

Of the large neutral amino acids in the ULF, glutamine, isoleucine, leucine, phenylalanine, tyrosine and valine exhibited similar trends in cyclic heifers with a decline in concentration on Day 16 of the estrous cycle. In pregnant heifers, concentrations of these amino acids as well as tryptophan were stable from Day 7 to Day 13, but increased (P<0.05) on Day 16 (glutamine) or Day 19 (isoleucine, leucine, phenylalanine, tryptophan, tyrosine, valine) of pregnancy. In contrast, concentrations of methionine decreased (P<0.05) from Day 13 onwards in cyclic heifers, while in pregnant heifers concentrations were lowest on Day 16.

### Expression of cationic amino acid transporters in the endometrium and conceptus during the peri-implantation period of pregnancy

**Figure 1:**
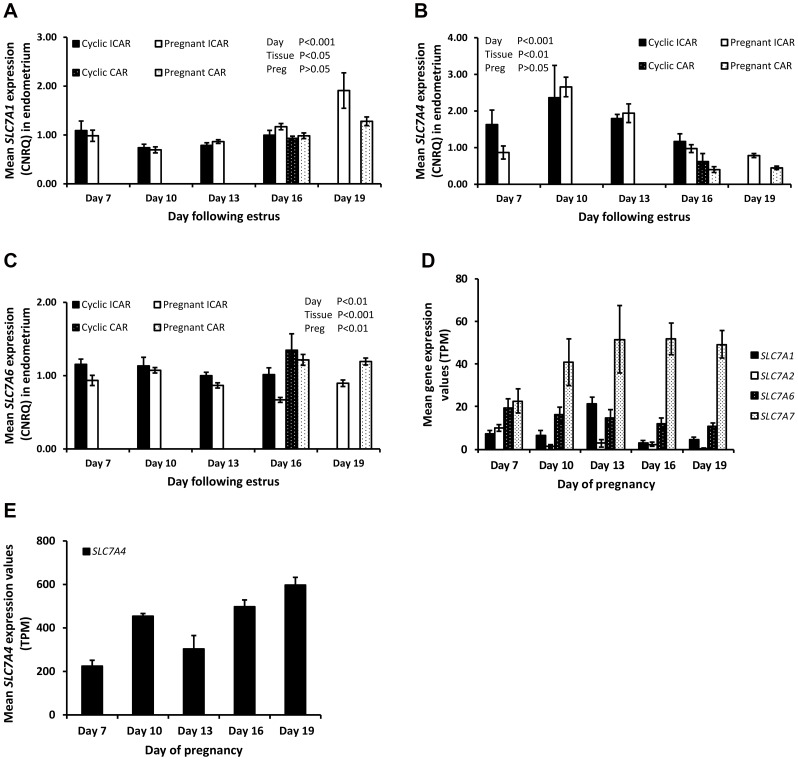
Gene expression values in the endometrium for cationic amino acid transporters as determined by qRT-PCR analysis. Data are displayed as mean calibrated, normalized, relative expression values in arbitrary units (CNRQ ± SEM) in the intercaruncular region of the endometrium from cyclic (solid bars) and pregnant (open bars) heifers and caruncular regions of cyclic (black bars white stipple) and pregnant heifers (while bars black stipple) (n = 5 per treatment per time-point). (A) *SLC7A1* CNRQ expression with a significant effect of day and tissue type (B) *SLC7A4* expression was significantly affected by day and tissue type and (C) *SLC7A6* whose expression was significantly affected by day, tissue type and pregnancy status. Significance was set at P<0.05. (D&E) Gene expression values for cationic amino acid transporters in the bovine conceptus during distinct developmental stages. Values were determined by RNA sequencing and are given as mean transcripts per million (TPM ± SEM) with n = 5 per time-point. A significant effect of stage of conceptus development was observed for all genes (P<0.05).

Endometrial expression of *SLC7A1* was affected by day with an increase (P<0.01) in expression at the latter stages of the estrous cycle and early pregnancy while all other members of this transport family (*SLC7A4* and *SLC7A6*) decreased (P<0.01) from Day 10 to 16 in the intercaruncular region of the endometrium (Table 2). In addition, expression of *SLC7A1* and *SLC7A4* mRNAs was less and *SLC7A6* expression was greater (P<0.05) in caruncular compared to intercaruncular regions of the endometrium (Figures 1A–C). Pregnancy significantly affected *SLC7A6* expression which was less abundant in intercaruncular regions of pregnant compared to cyclic endometria by Day 16. In the conceptus, *SLC7A4* was approximately 10-fold more abundant than the other cationic transporters. *SLC7A2* expression decreased with increasing conceptus age while *SLC7A6* and *SLC7A7* remained unchanged. The transporter *SLC7A1*, increased (P<0.01) as the conceptus developed from Day 7 to Day 13, but declined on Day 16 while *SLC7A4* expression was lower (P<0.01) on Day 10 compared to Day 7 (Figures 1 D&E).

**Table 2:** The effect of day, pregnancy status, progesterone concentration and/or caruncular v intercaruncular endometrial tissue on expression of solute like carrier family member genes for amino acid transporters in the embryo/conceptus and endometrium as determined by RNAseq and quantitative real-time PCR (qRT-PCR) analysis respectively.

Gene	Embryo/Conceptus	Endometrium			
	Day	Day	Caruncular v Intercaruncular	Pregnancy Status	P4 Concentration
*SLC1A1*	**	-	-	-	*
*SLC1A2*	NS	-	-	-	NS
*SLC1A3*	*	NS	***	NS	NS
*SLC1A4*	***	NS	***	NS	*
*SLC1A5*	***	***	NS	P = 0.06	*
*SLC38A11*	***	**	NS	NS	NS
*SLC38A2*	**	***	**	**	*
*SLC38A4*	-	***	NS	*	*
*SLC38A7*	*	***	***	NS	*
*SLC43A2*	***	***	***	NS	*
*SLC6A14*	NS	**	***	P = 0.06	*
*SLC7A1*	***	***	*	NS	*
*SLC7A2*	***	-	-	-	NS
*SLC7A4*	***	***	**	NS	NS
*SLC7A5*	**	**	NS	NS	*
*SLC7A6*	NS	**	***	**	NS
*SLC7A7*	NS	***	NS	NS	*
*SLC7A8*	P = 0.06	**	NS	NS	NS

Significant differences are noted by an astrisk (*). Significance set at P<0.05 (*), P<0.01 (**) or P<0.001 (***). A dashed line indicates that gene was not detectable in the specific tissue type.

### Expression of acidic amino acid transporters in the endometrium and conceptus during the pre-implantation period of pregnancy

**Figure 2:**
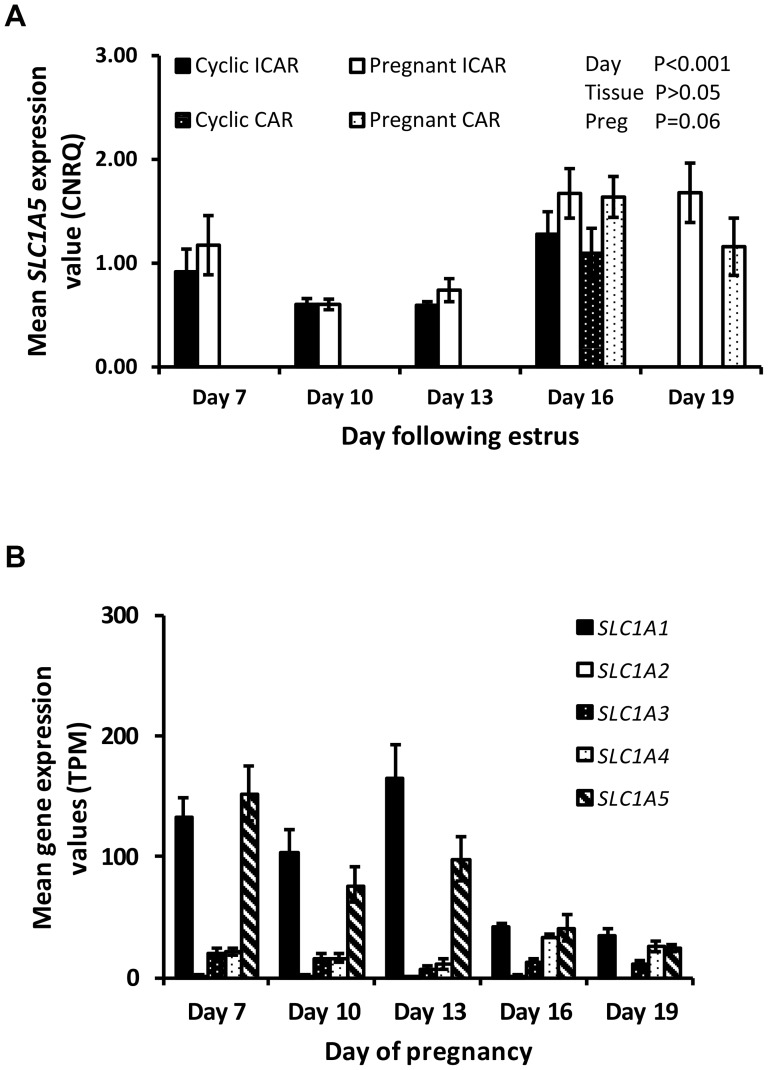
Gene expression values in the endometrium for acidic amino acid transporters as determined by qRT-PCR analysis. Data are displayed as mean calibrated, normalized, relative expression values in arbitrary units (CNRQ ± SEM) in the intercaruncular region of the endometrium from cyclic (solid bars) and pregnant (open bars) heifers and caruncular regions of cyclic (black bars white stipple) and pregnant heifers (while bars black stipple) (n = 5 per treatment per time-point). (A) *SLC1A5*CNRQ expression with a significant effect of day (P<0.001) and pregnancy status (P = 0.06). (B) Gene expression values for acidic amino acid transporters in the bovine embryo during distinct developmental stages. Values were determined by RNA sequencing and are given as mean transcripts per million (TPM ± SEM) with n = 5 per time-point. A significant effect of stage of embryo development was observed for all genes (P<0.05).

In the endometrium, the expression of *SLC1A1*, *SLC1A2 SLC1A3* and *SLC1A4* mRNAs was not affected by day. Expression of *SLC1A3* and *SLC1A4* mRNAs was lower (P<0.05) in caruncular compared to intercaruncular regions during the latter stages of the estrous cycle and early pregnancy (Table 2). The expression of *SLC1A5* in the endometrium increased (P<0.0001) as the estrous cycle and early pregnancy progressed and was affected by pregnancy status (P = 0.06: Figure 2A). In contrast, *SLC1A1*, *SLC1A3* and *SLC1A5* in the conceptus decreased with increasing conceptus age. The expression of *SLC1A4* increased (P<0.05) as the conceptus elongated while *SLC1A2* was not affected by stage of conceptus development (Figure 2B).

### Expression of neutral amino acid transporters in the endometrium and conceptus during the peri-implantation period of pregnancy

**Figure 3:**
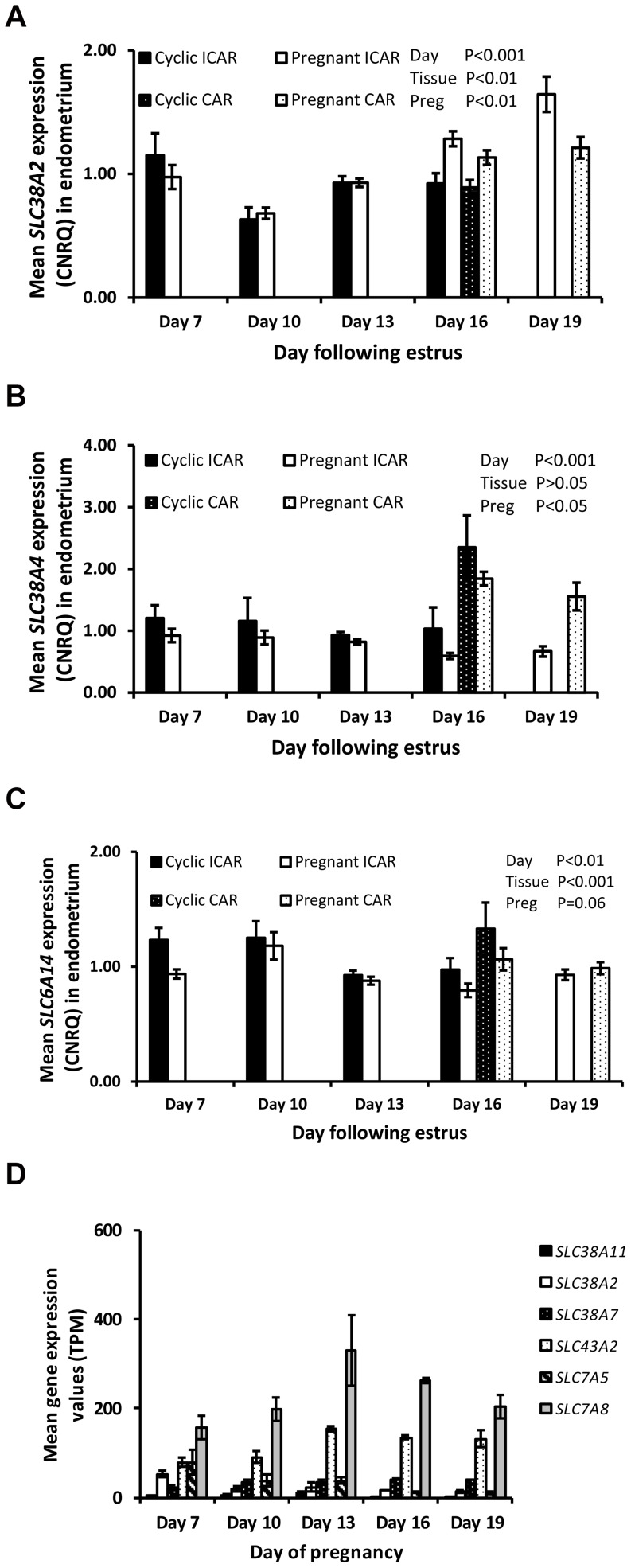
Gene expression values in the endometrium for neutral amino acid transporters as determined by qRT-PCR analysis. Data are displayed as mean calibrated, normalized, relative expression values in arbitrary units (CNRQ ± SEM) in the intercaruncular region of the endometrium from cyclic (solid bars) and pregnant (open bars) heifers and caruncular regions of cyclic (black bars white stipple) and pregnant heifers (while bars black stipple) (n = 5 per treatment per time-point). (A) *SLC38A2* expression with a significant effect of day, tissue type and pregnancy status (B) *SLC38A4* expression was significantly affected by tissue type and pregnancy status and (C) *SLC6A14* whose expression was significantly affected by day, tissue type and pregnancy status. Significance was set at P<0.05. (D) Gene expression values for neutral amino acid transporters in the bovine conceptus during distinct developmental stages. Values were determined by RNA sequencing and are given as mean transcripts per million (TPM ± SEM) with n = 5 per time-point. A significant effect of stage of embryo development was observed for all genes (P<0.05).

In the endometrium, there was an overall increase in expression of *SLC38A2* and *SLC43A2*, while *SLC38A7* and *SLC6A14* expression decreased as the estrous cycle and early pregnancy progressed (P<0.05). Expression of *SLC38A2*, *SLC38A7*, *SLC43A2*, and *SLC38A11* mRNAs was lower while expression of *SLC38A4* and *SLC6A14* mRNAs was greater (P<0.05) in caruncular compared to intercaruncular regions of the endometrium on both Days 16 and 19 of pregnancy. In pregnant heifers, *SLC38A2* expression increased from Day 10 to Day 19 while S*LC38A4* and *SLC6A14* expression was lower (P<0.05) in pregnant heifers on Day 16 compared to cyclic controls (Figure 3A–C). In contrast to the endometrium, expression of *SLC38A2* decreased, while *SLC38A7*, *SLC43A2*, *SLC38A11* increased (P<0.05) as conceptus development progressed (Figure 3D). Expression of *SLC6A14* was less than one transcript per million at all stages of conceptus development (data not shown).

Expression of the neutral amino acid transporter *SLC7A5* increased (P<0.01) in the endometrium as the estrous cycle and early pregnancy progressed with a co-ordinate decrease (P<0.05) in expression as the conceptus developed. Endometrial expression of *SLC7A8* decreased to Day 16 in both pregnant and cyclic heifers, but expression increased (P<0.05) on Day 19 in pregnant caruncular and intercaruncular tissue. *SLC7A8* in the conceptus was affected by day (P<0.05) with greatest expression on Day 13 of pregnancy.

### Modulation of amino acid transporters by progesterone in vivo

**Figure 4:**
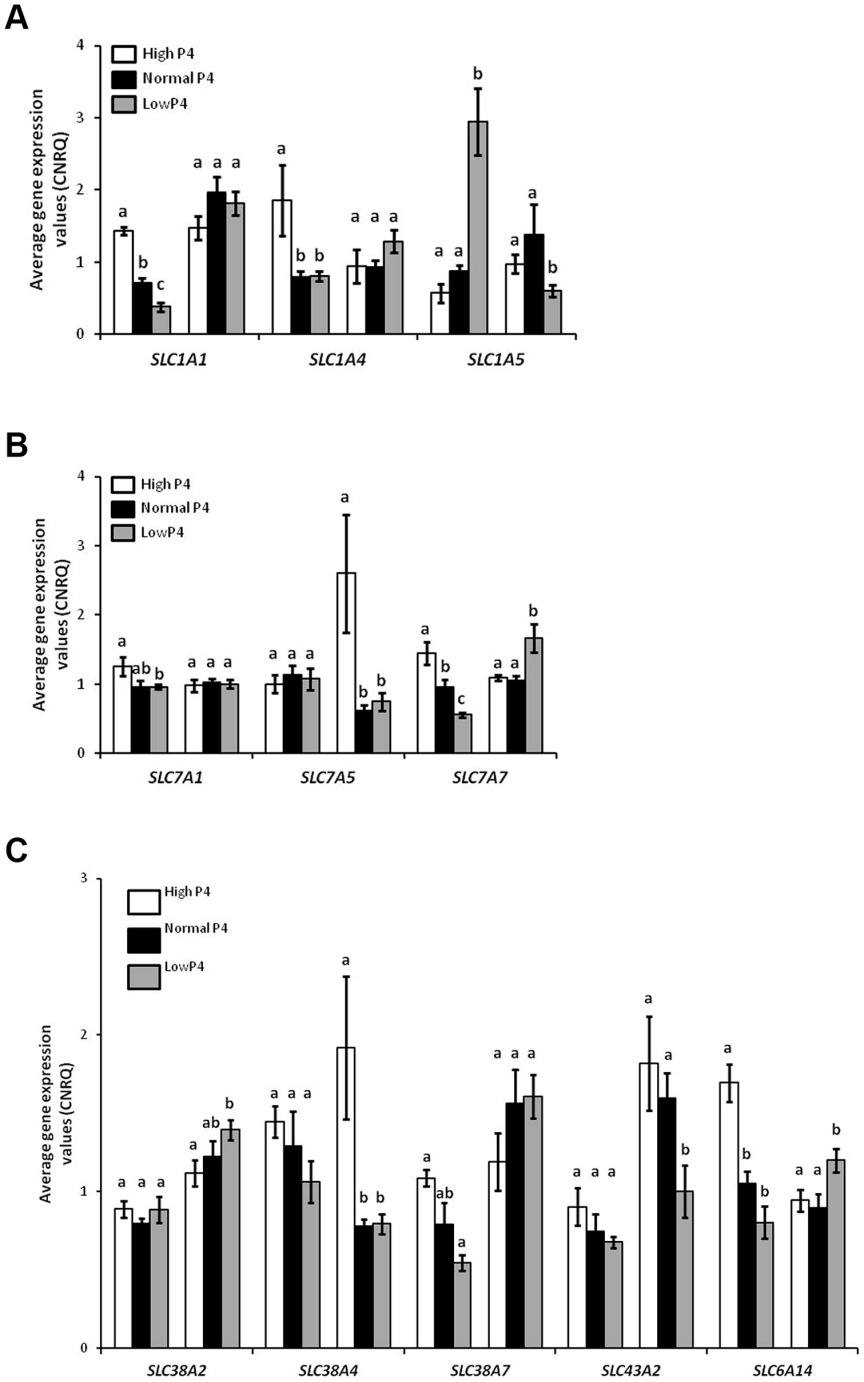
Gene expression values in the endometrium for heifers with high P4 (black bars: n = 5), normal P4 (white bars: n = 5) and low P4 (gray bars: n = 7) on Days 7 and 13 of the estrous cycle. All data are displayed as mean calibrated, normalized, relative expression values in arbitrary units (CNRQ ± SEM) for acidic amino acid transporters (A), basic amino acid transporters (B) and neutral amino acid transporter (C). Differences between treatments on a given day are denoted by a, b, c, when P<0.05.

Expression of three acidic amino acid transporters was modulated by concentration of P4 in vivo. On Day 7 of the estrous cycle, expression of *SLC1A1* and *SLC1A4* was higher (P<0.05) in high P4 heifers compared to control heifers and *SLC1A1* expression was lower (P<0.05) in the low P4 group (Figure 4A). In contrast, *SLC1A5* expression was higher (P<0.05) in the low P4 group on Day 7 compared to control heifers (Figure 4A). The cationic amino acid transporters *SLC7A1* and *SLC7A7* were more abundant on Day 7 in the high P4 group, while on Day 13, *SLC7A5* and *SLC7A7* were higher in the high and low P4 groups, respectively (P<0.05: Figure 4B). Manipulation of P4 concentrations had no effect on the expression of *SLC38A2*, *SLC38A4* or *SLC43A2* on Day 7 (P>0.05). However, P4 supplementation increased (P<0.05) *SLC38A7* and *SLC6A14* expression on Day 7. On Day 13 of the estrous cycle, heifers with low P4 had greater expression of *SLC38A2* and *SLC6A14*, while *SLC43A2* expression decreased as compared to values for control heifers (P<0.05: Figure 4C). The expression of *SLC38A4* was greater (P<0.05) in high P4 heifers while *SLC38A7* expression was similar amongst the three treatment groups.

## Discussion

This study is the first report on temporal changes in the amino acid content of ULF during the bovine estrous cycle and at key stages of the pre- and peri-implantation periods of pregnancy. Results of this study indicate that the expression of solute-like transporters responsible for the active transport of these amino acids from the endometrium into the ULF is distinct from the expression of transporters that move these nutrients into the conceptus. In addition, expression of certain amino acid transporters in the endometrium was modified by P4 and may therefore play a role in the the capacity of the uterus to support conceptus elongation.

In sheep, glycine was the most abundant amino acid detected in ULF followed by serine [13]. In the current study, threonine and glycine were the most abundant amino acids (10–20 fold greater than most other amino acids). Consistent with our study, data from sheep indicate that most amino acids increase after pregnancy recognition (e.g., arginine, histidine, glycine, glutamic acid, isoleucine, leucine, phenylalanine, tryptophan, tyrosine). In contrast, others do not follow the same pattern in both species; for example, threonine concentrations decreased on Day 16 and 19 of pregnancy in cattle but was reported to increase from Day 12–15 in sheep [13].Whether such discrepancies between cattle and sheep reflect true species differences or are artefacts of different technologies remains to be clarified.

The basic amino acids arginine and lysine are transported by the cationic transporters *SLC7A1*, *SLC7A2*, *SLC7A3*, *SLC7A4*, *SLC7A6*, *SLC7A7*, and *SLC3A1* while *SLC38A6* transports histidine [16]. Of these transporters expression of *SLC7A1* and *SLC7A2* increased in response to P4 supplementation and IFNT in vivo in sheep [16], coordinate with increased recoverable amounts of arginine in the ULF [13]. In this study, the overall abundance of arginine in ULF was similar during the estrous cycle and early pregnancy, but increased significantly on Day 19 of pregnancy. This was coordinate with increased expression of its transporter *SLC7A1* which also increased in the endometria of pregnant heifers on Day 19. In addition, *SLC7A1* expression was greater in heifers with high P4 on Day 7 which is a model associated with advanced conceptus elongation in heifers [30]. The presence of the conceptus was associated with increased expression of transporters for arginine in the endometrium resulting in the increased abundance of arginine in ULF available to the developing conceptus. Arginine was found to stimulate proliferation, protein synthesis and/or migration of sheep and pig trophoblast cells in vitro (Pigs [31]: Sheep [32]). Given that conceptuses of those species also elongate, it is likely that arginine plays a similar role in cattle. What is less clear in cattle, however, is the route of uptake of arginine given the decreased expression of members of the y+ family of transporters (*SLC7A1* and -*7A2*) as the conceptus elongates. It is possible that arginine is transported into the conceptus by other members of the cationic amino acid transporters e.g. *SLC7A6* and *SLC7A7* whose expression was maintained or increased as the conceptus was undergoing elongation.

The acidic amino acids glutamic and aspartic acid are transported by *SLC1A1*, -*1A2*, -*1A3* and other acidic amino acids are transported by *SLC1A4* and -*1A5* from the ASC transport system [33]. Both aspartic and glutamic acid increased between Days 16 and 19 of pregnancy. There was no difference on Day 16, the day of pregnancy recognition [2], [3]; however, endometrial expression of *SLC1A5* increased in pregnant heifers on Day 16 and was maintained to Day 19. It is likely that the increase in these amino acids on Day 19 and not before is due to a lag between the increased expression of their transporters on Day 16 and the subsequent detection of an increased abundance of the amino acids. Although concentrations of these acidic amino acids increase in bovine ULF by the initiation of implantation (Day 19), the mechanism of transport of these amino acids seems to differ between the endometrium and conceptus. Given that *SLC1A5* increased in the endometrium with a corresponding increase in *SLC1A4*, we propose that the transport of glutamic and aspartic acid into the uterine lumen is mediated predominantly via *SLC1A5*; however, uptake of these amino acids by the conceptus is via *SLC1A4* in cattle. In addition, the early increase in *SLC1A5* expression in P4-supplemented heifers suggests that advanced conceptus elongation in this model is, in part, driven by increased transport of both aspartic and glutamic acids into the uterine lumen via increased expression of *SLC1A5* and not other members of the acidic amino acid transporter family.

The most abundant amino acids in bovine ULF in this study were the neutral amino acids. Of the neutral amino acid transporters analysed in the endometrium, increased expression of *SLC38A2* in the pregnant endometrium on Days 16 and 19 suggests that this gene is most likely involved in the transport of neutral amino acids into ULF during the pre-implantation period of pregnancy in cattle. Interestingly, asparagine and threonine were less abundant in ULF of pregnant compared to cyclic heifers on Days 7 and 10, respectively. This seems at odds with the fact that pregnancy recognition, has not occurred, and previous studies have shown that Days 15 and 16 of pregnancy are the earliest that differences in gene expression in the endometrium are detectable between cyclic and pregnant heifers [34], [35].

Altering circulating concentrations of P4 in vivo can either advance (in the case of high P4 [36]) or delay (in the case of low P4 [29]) conceptus elongation following transfer of a blastocyst to heifers on Day 7 of the estrous cycle. This study clearly demonstrates that amino acids are an important component of ULF during the estrous cycle and early pregnancy and that the transport of these molecules into ULF from the endometrium occurs throughout this period. An early increase in P4 concentrations increased endometrial expression of *SLC1A5*, *SLC38A7*, *SLC6A14*, *SLC7A1* on Day 7 post-estrus, with an early (Day 7) and sustained increase in expression to Day 13 for *SLC38A4*, *SLC7A5* and *SLC7A7* compared to control heifers, suggesting that one way in which conceptus elongation is advanced, is through effects on the maternal amino acid transport system. Conversely, in heifers with a delay in the post-ovulatory increase in P4 (associated with smaller conceptuses), expression of *SLC43A2*, *SLC6A14*, *SLC7A6* and *SLC7A7* for amino acid transport into the ULF was sub-optimal. These P4-regulated changes in expression of the transporters in the endometrium are consistent with lower concentrations of histidine and asparagine in ULF of low P4 heifers on Day 13 [37]. Therefore, in vivo manipulation of P4 alters the expression of genes for amino acid transporter from the endometrium into the ULF which has clear consequences for conceptus elongation in vivo which supports findings from studies with ewes [38].

Previous studies have demonstrated that in vitro derived blastocysts have a higher amino acid turnover than their in vivo derived counterparts and, overall, expanded blastocysts deplete more amino acids than those that do not undergo expansion [23]. This is interesting in the context of this study in which the amount of detectable amino acids in ULF increased as development of the blastocyst progressed to an elongated filamentous conceptus. However, once hatched from the zona pellucida the substantial increase in the composition of specific amino acids in the ULF suggests increased requirements for these amino acids to drive conceptus elongation. Moreover, substantial increases in the abundance of amino acids in the gravid as compared to the non-gravid uterine horn on Day 18 of pregnancy [15] indicate that the presence of the conceptus stimulates transport of amino acids into the uterine lumen. Interestingly, the amounts of amino acids in ULF were reduced in heifers with a developmentally compromised conceptus (i.e. cloned embryos) [39] and in sub-fertile animals [40]. The fact that we demonstrated a significant increase in the amount of amino acids in ULF on Day 19 of pregnancy, along with increased expression of their transporters in the endometrium, is consistent with the notion that increased abundance of amino acids in ULF is required for successful pregnancy establishment in cattle. In conclusion, results of this study demonstrated that most amino acids increase in ULF between Days 16 and 19 of pregnancy which is after pregnancy recognition has occurred. The amino acid transporters are temporally regulated in a tissue-specific manner in the endometrium and conceptus during the peri-implantation period of pregnancy and we propose that the transport mechanisms for amino acids into ULF from the endometrium are distinct from those of the conceptus. Moreover, expression of the amino acid transporters in the endometrium in vivo under conditions where conceptus elongation is advanced (elevated P4) or retarded (low P4), may alter the transport of acidic, neutral and cationic amino acids into ULF. We propose that transport of amino acids into the uterine lumen contributes to the capacity of the uterus to stimulate elongation of the conceptus during the peri-implantation period of pregnancy in cattle.

## Supporting Information

Table S1 **Primer information used for quantitative real time PCR analysis of candidate genes.** All primers were used at a concentration of 300 nM in a final reaction volume of 15 µl. A dissociation curve was included to ensure specificity of each primer pair.
(XLSX)
